# Inspectors, Examiners, Censors

**Published:** 1872-04

**Authors:** Thos. K. Beecher


### INSPECTORS, EXAMINERS, CENSORS.

T. K. BEECHER.

A vague rumor reaches me that an attempt is making at Albany to defend the community against quacks and charlatans, by establishing boards of competent examiners to ascertain the ability or disability of the men who practice medicine and surgery among their fellows.

This movement causes a flurry among the doctors, and wakens anew the war of the "schools"—the regulars, homeopaths, hydropaths, eclectics, and the free doctors. I name them in the order of their numbers. Shall the examiners be *regulars?* Where then shall the able and successful practitioner of other names look for appreciation and reward? Shall they be homeopaths? Never! never! indignantly reply the regulars. Shall they be half and half? Absurd and impracticable! Every meeting would result in a dead-lock.

Shall our ignorant citizens, racked by pain or scared by death's shadow, be allowed to fall victims to bold quacks, charlatans and medical adventurers? That is indeed pitiable. But what can be done about it?

The age is impatient of control, and clamoring for freedom. May not a doctor practise as he pleases? May not every man choose his own doctor as he does his own minister? If we have outlived ecclesiastical tyranny and outgrown the Pope, shall we elect a close corporation of medical tyrants, and smother all genius that would advance the healing art, by giving legal authority to a set of old fogy, hide-bound doctors to oppress and exterminate their young rivals?

So run the arguments and appeals on one side and the other. They have set me a thinking, and here are the thoughts.

———The useful and lawful industry of citizens may be classed in two: (1.) The industries that use man himself as the material on which they work, and (2.) The industries that expend themselves upon his surroundings, and not upon the man. For instance:

A physician benefits or damages the man himself; but the architect and carpenter work upon the house only. A school master takes raw children to drill them into intelligence, good manners and morals; but a tailor takes the boy and merely clothes him. The bungling of a doctor or school master damages the man himself who submits to treatment; but the bungling of the carpenter or tailor damages a man's circumstances only.

Among the men who *directly* harm or help their fellows, I may enumerate: Druggists, liquor dealers, grocers, market-men, bakers, cooks, school masters, clergymen, physicians, surveyors, hotel-keepers, printers—booksellers and others like. They all take their fellow man and do for him a work that affects him personally.

As samples of the more numerous second class I name: Carpenters, blacksmiths, shoemakers, saddlers, day-laborers, dress-makers, tailors, lawyers, hardware men, jewelers and shopkeepers generally. These and such supply the wants of their fellow man but do not act directly upon the man himself.

The men belonging to the first class are the ones over whom it has usually seemed best to establish examiners and inspectors. True, in the olden time, government meddled with pretty much everything done by man. But these industries enumerated are the ones most recently set free from the supervision of governmental agents.

Why is this? I answer, because in every deal of the citizen with men of the first class, he is called upon to submit himself to them blindly, and take the very serious consequences. A man must trust his baker, his butcher, his priest, his tapster, his teacher and his doctor.—He is a layman, they are initiated. Lest they damage their fellow man, government steps in, and by appointed experts examines the ones who desire to be trusted, and delivers society from bunglers and knaves by refusing license.

I suppose that public health and morals are suffering to-day as much from the adulterations of beer, wine and strong drink as from any one cause that can be named. Yet what county can name a citizen so strong in science, in honesty, in courage and public confidence, that he can be safely armed with power to examine and confiscate all the deleterious drink that he can lay hands upon? He would be bought up or howled down in less than a month.

In all our large cities there are inspectors of meat and fish. Nevertheless, sick beef and stale fish can be bought by the ton in broad daylight.

The fact is, that under a democratic government there are so many masters, and so few of hem know anything for certain, that government itself is becoming a mere sham. There is not a land under the sun with as many laws as we have, and none also so nearly lawless.—"That's against the law, sir!" "Oh, law be d—d—Who cares for law!"

He who proposes to regulate or restrain any powerful wrong by force of law, shows himself quite untaught by the signs of the times.—

The laws that seem to have force remaining are relics of an ancient authority, that have not as yet been swashed and rotted and swept away by the tides of freedom rolling in from the great ocean of lawlessness. I call to mind no new law, imposing restraints or duties upon citizens that has not been and is not successfully evaded or resisted. And as a general rule, public sentiment is on the side of any bold offender who defies the law. No greater help can be given to a showy quack in medicine or a noisy sciolist in theology than to attempt to repress him by law. He has only to fling his flag to the breeze, shout freedom, and he has the people at his back, and himself a hero leading the van-guard of Progress!

Well! What of it? I answer, that when the great ship goes to pieces look to your boats, and when the boats fail, look out, every man, for a life preserver, and strip for a swim—then wait for help.

When the great catholic Church is rent with schism, let the resulting denominations win trust and honor by well deserving. When ecclesiastical bodies degenerate into mere party caucuses, planning church extension rather than cultivating true religion, let the pious man put faith in God, gird himself and wait. By and by his personal and persistent worth will be recognized.

The same is true in medicine and surgery.—When medical schools equip their graduates with witty flings and prejudices against rival schools; when county and state societies degenerate and become shelters for regular ignorance, and armed forts against irregular science and skill; when doctors oppose rivals more passionately than they do disease;—then let the self-respecting, honest practitioner fall back upon first principles and win reputation and success by deserving them. Let him stand still in one place, industrious, observant and responsible.—Let the charlatans and nostrum-venders come and go, leaving their fleeced and enfeebled victims behind them. Let him avoid strife and give himself wholly to his work.

Ere many years the worth of his personal qualities, and his well-earned fame, will stand him in better stead than acres of diplomas and official license. By their fruits ye shall know them. Quacks and charlatans must keep moving.—He alone is trustworthy who stands by his work, ready to acknowledge his failures or wear the laurels of success with the beautiful composure of an honest man who has done his best.

———Much as we need inspectors, examiners and censors, we cannot have them. Their day has gone by. Personal and professional character and responsibility are our only remaining safeguards against charlatanry. There will always be fools enough for the quacks to experiment upon. Let wise men wait and see if the new comer kills or cures his fools! See whether he fathers his own work. If he shows himself trustworthy, he ceases to be a quack, and proves himself a true physician. Having proved him, trust him, no matter what school he belongs to in medicine, or denomination in religion.

---

THE man who said two porcupines make one prickly pair, is a knight of the quill, and not a professor of the higher mathematics.

